# An electrical swinging!

**DOI:** 10.1093/ehjcr/ytae472

**Published:** 2024-09-23

**Authors:** Swasthi S Kumar, Sudipta Mondal, Jyothi Vijay

**Affiliations:** Department of Cardiology, Electrophysiology Division, Sree Chitra Tirunal Institute for Medical Sciences and Technology, Thiruvananthapuram, Kerala PIN 695011, India; Department of Cardiology, Electrophysiology Division, Sree Chitra Tirunal Institute for Medical Sciences and Technology, Thiruvananthapuram, Kerala PIN 695011, India; Department of Cardiology, Electrophysiology Division, Sree Chitra Tirunal Institute for Medical Sciences and Technology, Thiruvananthapuram, Kerala PIN 695011, India

## Case

**Figure 1:**
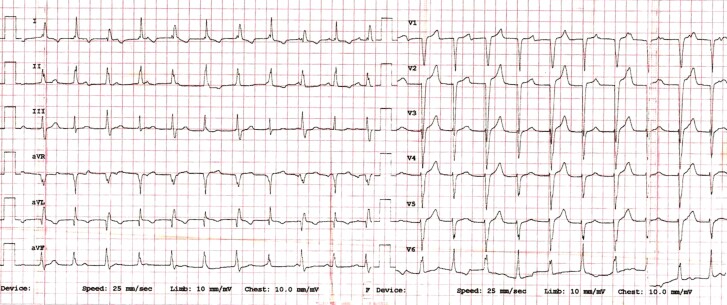
Twelve-lead electrocardiogram of the patient during tachycardia (see text for explanation).

A 35-year-old patient with a perimembranous ventricular septal defect was electively planned for intracardiac repair. Following surgery, he required inotropes for haemodynamic support. However, the post-operative electrocardiogram showed a wide complex tachycardia (*Figure 1*, Supplementary material online, *Figure S1*). Despite amiodarone infusion and electrical cardioversion, the patient remained in tachycardia and there was no termination and re-initiation as well.

### Question 1

#### Which of the diagnoses is possible in this case?

- Atrioventricular nodal re-entrant tachycardia (AVNRT)
- Orthodromic atrioventricular reciprocating tachycardia (AVRT)
- Antidromic AVRT
- Junctional ectopic tachycardia (JET) or Ventricular tachycardia (VT) with electrical alternans
- JET or VT with cycle length alternans

#### Answer: D

This electrocardiogram of regular wide complex tachycardia (WCT) with a QRS duration of 120 ms with atrioventricular dissociation (*Figure 1*, Supplementary material online, *Figure S1*) rules out all AV node-dependent regular tachycardia like AVNRT, AVRT, and atrial tachycardia or atrial flutter. There were no cycle length variations. The QRS complex exhibited alternating morphology, consistent with variable degrees of left bundle branch block patterns. This finding suggests the potential presence of underlying distal conduction system disease. Electrical alternans can also be observed in the mechanical swinging of the heart within the pericardial cavity. However, in such cases, the QRS complex morphology remains unchanged with a reduction in amplitude, unlike the presented case. Although the first differential diagnosis of WCT in structural heart disease is VT, a typical post-operative state, refractoriness to medical treatment and instant resumption of tachycardia following electrical cardioversion (suggesting automaticity rather than re-entry), all pointed towards a JET with electrical alternans rather than a VT. Given the absence of dropped QRS and constant R–R intervals, the possibility of ectopic atrial tachycardia with AV Wenckebach is also unlikely.

### Question 2

#### Which electrocardiographic presentation is extremely unlikely in JET?

- Narrow complex tachycardia (NCT) with 1:1 VA relation
- NCT with VA dissociation
- Wide complex tachycardia (WCT) with VA dissociation
- WCT with bigeminy
- Irregularly irregular NCT with VA dissociation

#### Answer: E

Junctional ectopic tachycardia is a well-recognized complication following congenital heart surgery, particularly arising within the initial 72 post-operative hours. This arrhythmia is most frequently observed after procedures involving perinodal tissues.^1^ Potential contributing factors include direct mechanical trauma or indirect stretch injury to perinodal tissues. ECG manifestations typically include narrow complex tachycardia with AV dissociation, although occasional cases of 1:1 VA conduction may also be observed. In less frequent presentations, JET may manifest with right bundle branch block (RBBB) (post-operative RBBB in Tetralogy of Fallot repair) or VA Wenckebach. Junctional ectopic tachycardia presenting as WCT with echo-bigeminy has also been reported in infants.^2^ Overdrive pacing or administration of Adenosine may help in confirming the diagnosis.

### Question 3

#### Which of the following is unlikely to be effective in post-operative JET?

- Therapeutic cooling
- Amiodarone
- Ivabradine
- Beta-blockers
- Nifekalant

#### Answer: D

Junctional ectopic tachycardia typically demonstrates refractoriness to medical therapy and electrical cardioversion. Fortunately, the majority of JET episodes are self-limiting. Therapeutic hypothermia with a target core temperature range of 32–34°C, atrial overdrive pacing to maintain AV synchrony, amiodarone, and ivabradine are treatment options that may be employed for JET.^3^ In rare instances and based on limited case reports, alpha-2 adrenergic agonists, flecainide, propafenone, procainamide, sotalol, or nifekalant, may be considered. Digoxin, beta-blockers (except sotalol), and calcium channel blockers are unlikely to be effective. Catheter ablation is reserved as a last resort for patients with JET who experience haemodynamic compromise despite maximal medical therapy.

## Supplementary Material

ytae472_Supplementary_Data

## Data Availability

Data are available on request from authors.

## Supplementary material

**Consent:** Obtained from the patient in line with COPE guidance.

**Funding:** None declared.
