# Supplementary figures and images for: An electrical swinging!
Source: Eur Heart J Case Rep. 2024 Sep 23;8(9):ytae472. doi: 10.1093/ehjcr/ytae472 (PMC11417611; doi:10.1093/ehjcr/ytae472)

## Slide 1
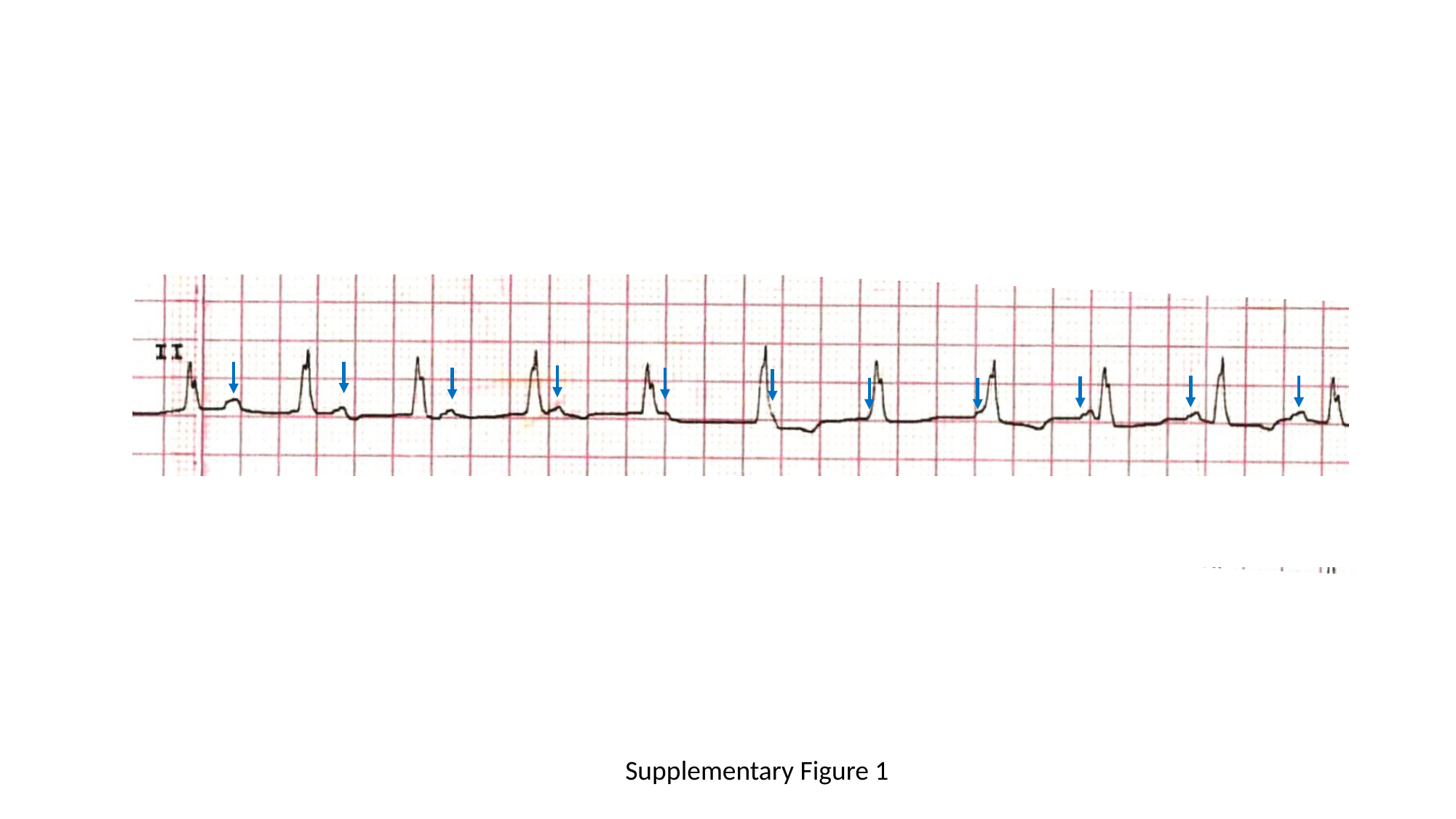

Supplementary Figure 1

Supplement: ytae472_Supplementary_Data [file ytae472_supplementary_data.pptx]
